# Special Issue: Silicon Carbide: From Fundamentals to Applications

**DOI:** 10.3390/ma14051081

**Published:** 2021-02-26

**Authors:** Sergey Kukushkin

**Affiliations:** Institute for Problems in Mechanical Engineering of the Russian Academy of Sciences (IPME RAS), 199178 Saint-Petersburg, Russia; sergey.a.kukushkin@gmail.com; Tel.: +7-(812)-321-47-84

Most of the wide variety of electronic devices today are silicon-based. Silicon was and still remains the main material in the electronics industry. Modern life, however, requires more and more variety of devices and instruments, which cannot be created based on silicon alone.

Modern life and the market demand the creation of Light Emitted Diods (LEDs), semiconductor lasers, high-electron mobility transistors (HEMT), gas control sensors and detectors, microwave devices, new generation of pyro and piezoelectric sensors, optical switches, devices emitting and receiving terahertz radiation, etc. Recently, an urgent need has arisen for both LEDs emitting hard ultraviolet (UV) radiation and UV detectors.

In this regard, an intensive search for other materials is currently underway that can, if not completely, but at least partially, replace silicon. Silicon carbide (SiC) is one of such materials.

Silicon carbide is the only binary compound of silicon and carbon that exists in the solid phase under normal conditions. As early as 1824, Jöns Jakob Berzelius first suggested that a chemical bond might exist between silicon and carbon. Silicon carbide is rare in the Earth environment, but it is widespread in the universe, its inclusions are often found in meteorites. The first SiC crystals of extraterrestrial origin were discovered by Henry Moissan in 1905 during the examination of meteorites in the Devil’s Canyon in the Arizona desert. In honor of him, the mineral was called moissanite. Producing artificial silicon carbide was first patented in 1891 by Edward Acheson. Ironically, the active use of silicon carbide in microelectronics began only in recent years, despite the fact that silicon carbide is one of the first materials of solid-state electronics. As early as 1907, H. Round observed luminescence when an electric current passed through a SiC crystal. In 1923–1940, Oleg Losev investigated the electroluminescence of silicon carbide in more detail. Losev also found a relation between current rectification and electroluminescence in SiC. Thus, the two most important phenomena for semiconductor electronics—electroluminescence and the rectifying properties of p-n structures—were first discovered in SiC crystals. SiC crystals have a large bandgap in comparison with Si and GaAs, which allows a significant expansion of the operating temperatures of electronic devices (theoretically up to ~1000 °C). Due to the larger (by order of magnitude) breakdown field of SiC than that of silicon, the doping level of a SiC diode can be two orders of magnitude higher than that of a silicon diode at the same breakdown voltage. Silicon carbide is a radiation-resistant material. The high thermal conductivity of SiC (at the level of thermal conductivity of copper) greatly simplifies the problem of heat removal from devices. This property, combined with high permissible operating temperatures and high saturation rates of carriers (high saturation currents of field-effect transistors), makes SiC devices very promising for use in power electronics. In addition, the high Debye temperature, which determines the temperature at which phonons arise, indicates the high thermal stability of SiC. Thus, silicon carbide surpasses classical semiconductor materials, Si and GaAs, in almost all-important criteria.

The topic of this issue covers a range of questions devoted to the study of fundamental and applied aspects of silicon carbide. The issue includes seven articles devoted to both the properties of silicon carbide and the possibility of creating various electronic devices and sensors on its basis.

Below, we provide a brief summary of the works included in the collection. Three of the total seven articles [1,2,3] are devoted to the technology of creating electronic devices based on silicon carbide single crystals. So, in Reference [1], a comparative study of the electrical and thermal parameters of three commercial Schottky diodes 1200 V/5 A MPS (Merged PiN Schottky) made on 4H-SiC was carried out. The authors demonstrated that to create high-quality SiC-based diodes, an optimal combination of good electrical forward conductivity with good heat dissipation is necessary. The authors have shown that a specially created design, which is described in detail in this article, allows well dissipation of the heat generated during the operation of the diode, and significantly increases the throughput of pulsed currents of diodes of this type. In Reference [1], it was demonstrated that high bipolar electrical conductivity causes overheating of the device, and the design of the device with a low thermal resistance can accelerate heat removal and limit the junction temperature during voltage surges. The authors cite in Reference [1] a description of the MPS diode design which can provide 60% higher pulse current density compared to other technologies.

Reference [2] is devoted to the creation of neutron detectors based on SiC. The authors noted that the use of sensors for neutrons based on SiC single crystals is expanding more and more. Devices of this kind are small in size and show high radiation resistance. SiC neutron detectors can be used in new generation neutron welding machines. Silicon carbide is highly resistant to neutron radiation, devices based on it have a short response time, and it itself is durable and resistant to destruction by high-energy radioactive particles. The authors of Reference [2] showed that the absence of polarization effects under neutron and alpha irradiation makes silicon carbide an alternative to diamond sensors for detecting of fast neutrons.

The authors of Reference [3] present a new development of a step-recovery diode (DSRD) based on 4H-SiC. This diode is a kind of open-type pulse diode based on wide-bandgap material. The authors of this work were the first to introduce a super-junction (SJ) structure into a SiC DSRD design. In their opinion, this leads to an increase in the rigidity of the recovery process, and at the same time, improves its blocking ability. The paper shows that the use of a diode with the SJ results in higher voltages, higher breaking currents, and more severe recovery characteristics than conventional SiC DSRDs.

References [4,5] can be attributed to the second group of works, which describe technologies that improve the properties of both ceramics based on silicon carbide and the properties of nanostructures made of silicon carbide. The authors of Reference [4] added carbon and B4C to create ceramics with high density. In doing so, they used the method of spark plasma sintering at a temperature of 1950 °C with the simultaneous creation of a pressure of about 50 MPa for 5 min. Carbon was added by the authors of Reference [4] to remove oxygen from the SiC ceramic. It turned out that the addition of about 1.5 wt.% C leads to almost complete removal of the oxide layer from SiC. As a result of these operations, the mechanical properties of the SiC ceramics were dramatically improved. The highest hardness and elasticity values of the modulus were 27.96 and 450 GPa, respectively. The research results have shown that if the ceramic is made of the 6H-SiC polytype, then during its sintering with C and B4C, small coaxial grains grow. When one uses 4H-SiC, a similar process causes the growth of large coaxial grains.

Recently, studies of the process of formation and growth of whisker nanocrystals, nanowires, and other objects of this kind have been of great interest. As a rule, nanocrystals grown on the basis of GaAs, GaN, and InN compounds are being studied. There are not many studies devoted to the growth of SiC-based nanowires. Published in this issue, Reference [5] reports on the growth of SiC nanowires on a single crystal Si substrate by pyrolysis of polycarbosilane and using two catalyst films (Al_2_O_3_ and Ni) of different thicknesses (2, 4, and 6 nm). Catalyst films were deposited on a Si substrate, and SiC nanowires were grown by two mechanisms: the growth mechanism using oxides and the vapor-liquid-solid mechanism. As a result, pearl-chain SiC nanowires and straight SiC nanowires were obtained. The prepared nanowires exhibit excellent photoluminescent properties, the emission spectra exhibit two emission peaks at 395 and 465 nm, and have good thermal stability below 1000 °C. The experimental results showed that the catalysts Al_2_O_3_ and Ni play an important role and influence on the morphology and properties of nanowires.

Possible applications of SiC substrates to obtain graphene sensors for biological applications are described in Reference [6]. The authors of this work grew graphene films on a semi-insulating 6H-SiC substrate by thermal decomposition at a temperature of ~1700 °C. Note that the method of thermal destruction of the silicon carbide surface makes it possible to obtain graphene films with a sufficiently high structural perfection. The use of semi-insulated SiC substrates makes it possible not to transfer the grown graphene onto a dielectric substrate. The presence of such electrophysical parameters of graphene, as the maximum area-to-volume ratio, low carrier concentration in combination with their high mobility, makes it possible to obtain supersensitive resistive sensors on its basis. The practical application of graphene gas sensors is hindered by the lack of selectivity when registering various gases. The use of an antigen–antibody pair allows one to solve the problem of selectivity of biosensors and opens up very wide possibilities for the use of sensors based on graphene in medicine and biology. This approach can lead to the creation of portable biosensors capable of detecting diagnostically significant markers of diseases in biological fluids in the express analysis mode.

In Reference [7], authors describe unusual, anomalous properties of the interface between the epitaxial 3C-SiC layer and the silicon substrate Si, formed during the growth of 3C-SiC films on silicon by a new method that is fundamentally different from standard methods. The essence of the growth method described in Reference [7] consists in the self-consistent substitution of a part of Si atoms by carbon atoms, which is carried out in the near-surface layer of the Si substrate using the chemical reaction of interaction of carbon monoxide with solid silicon according to the formula:

2Si (crystal) + CO (gas) = SiC (crystal) + SiO (gas) ↑(1)

The authors of Reference [7] showed that the synthesis of SiC by this method makes it possible to avoid the formation of lattice mismatch dislocations and to obtain SiC films of very high quality. This method is fundamentally different from the standard methods of SiC growth, in which SiC precursors are being deposited onto the substrate surface from the external environment. When SiC is grown by this method, growth occurs inside the Si substrate layer. The term “self-consistent” means that the removal of the Si atom from the lattice and the incorporation of the C atom in its place occur simultaneously. In this case, the oxygen atom plays the role of a catalyst for the substitution reaction. As a result of this type of transformation of Si into SiC, huge elastic stresses, of the order of 250 GPa, arise at the film–substrate interface. These stresses lead to the breaking of some of the chemical bonds between Si and SiC. In this case, silicon atoms from the substrate are attracted to the interface located on the side of the SiC film. As a consequence of this attraction, a thin intermediate layer with a dielectric constant corresponding to a semimetal is formed at the 3C-SiC (111)/Si (111) interface. The properties of the 3C-SiC (111)/Si (111) interface corresponding to the minimum energy have been calculated using quantum chemistry methods. Calculations have shown that Si atoms in silicon carbide at the interface, which are as far as possible from the Si atoms of the substrate and do not form a chemical bond with them (there are only 12% of them), provide a sharp peak in the density of electronic states near the Fermi energy level. As a result, the interface acquires the properties of a semimetal. Thus, in this work, it is shown that SiC films grown on Si will have a number of unique electrophysical and magnetic properties.
